# Evaluation of Web-Based Consumer Medication Information: Content and Usability of 4 Australian Websites

**DOI:** 10.2196/ijmr.5651

**Published:** 2016-07-21

**Authors:** Magdalena Z Raban, Amina Tariq, Lauren Richardson, Mary Byrne, Maureen Robinson, Ling Li, Johanna I Westbrook, Melissa T Baysari

**Affiliations:** ^1^ Centre for Health Systems and Safety Research Australian Institute of Health Innovation Macquarie University Sydney Australia; ^2^ School of Public Health and Social Work Faculty of Health Queensland University of Technology Brisbane Australia; ^3^ Healthdirect Australia Sydney Australia; ^4^ Department of Clinical Pharmacology and Toxicology St Vincent's Hospital Sydney Australia

**Keywords:** consumer health information, health communication, prescription drugs, nonprescription drugs, drug information service, Internet, usability testing

## Abstract

**Background:**

Medication is the most common intervention in health care, and written medication information can affect consumers’ medication-related behavior. Research has shown that a large proportion of Australians search for medication information on the Internet.

**Objective:**

To evaluate the medication information content, based on consumer medication information needs, and usability of 4 Australian health websites: Better Health Channel, myDr, healthdirect, and NPS MedicineWise .

**Methods:**

To assess website content, the most common consumer medication information needs were identified using (1) medication queries to the healthdirect helpline (a telephone helpline available across most of Australia) and (2) the most frequently used medications in Australia. The most frequently used medications were extracted from Australian government statistics on use of subsidized medicines in the community and the National Census of Medicines Use. Each website was assessed to determine whether it covered or partially covered information and advice about these medications. To assess website usability, 16 consumers participated in user testing wherein they were required to locate 2 pieces of medication information on each website. Brief semistructured interviews were also conducted with participants to gauge their opinions of the websites.

**Results:**

Information on prescription medication was more comprehensively covered on all websites (3 of 4 websites covered 100% of information) than nonprescription medication (websites covered 0%-67% of information). Most websites relied on consumer medicines information leaflets to convey prescription medication information to consumers. Information about prescription medication classes was less comprehensive, with no website providing all information examined about antibiotics and antidepressants. Participants (n=16) were able to locate medication information on websites in most cases (accuracy ranged from 84% to 91%). However, a number of usability issues relating to website navigation and information display were identified. For example, websites not allowing combinations of search terms to be entered in search boxes and continuous blocks of text without subheadings.

**Conclusions:**

Of the 4 Australian health information websites tested, none provided consumers with comprehensive medication information on both prescription and nonprescription medications in a user-friendly way. Using data on consumer information needs and user testing to guide medication information content and website design is a useful approach to inform consumer website development.

## Introduction

Medication is the most common intervention in health care [1]. A 2010 survey of 12,262 consumers revealed that approximately 80% of Australians sought health information on the Internet, and of these individuals, approximately 70% sought information on medication [2]. Focus groups with Australian consumers showed that consumers viewed the Internet as an important source of medication information, but also that consumers varied in their search and appraisal skills [3]. Examining written medication information available on the Internet is of value as this information has the potential to affect consumers’ medication taking behavior and satisfaction [4,5].

Evaluations of Web-based health information have typically utilized instruments with various criteria [6] covering technical details (eg, disclosure of authorship and sponsorship, provision of references) [7], design features (eg, layout, speed), readability (eg, Flesch Reading Ease, Simple Measure of Gobbledygook (SMOG)) [8,9], accuracy, and completeness of information. However, many of the criteria lack validity and reliability and have certain gaps [6,10-12]. For example, a 2002 systematic review of the criteria used to evaluate health websites identified user testing as a neglected area [6]. More recently, user testing has been used to assess websites in a number of studies and has highlighted the importance of this approach in usability evaluation [13,14]. For example, 1 study used 4 rounds of user testing to improve an Internet-based hemophilia self-management tool for adolescents [13]. Another study used user testing and identified the need for websites to take user age into account in their design [14].

Although there have been numerous evaluations of Web-based health information [6,10], fewer studies have evaluated Web-based medication information [3,9,15-22]. Given the importance of Web-based medication information to consumers [23], this study aimed to evaluate the medication information on 4 frequently used Australian websites. The evaluation took a unique approach by evaluating both website content and usability and by being guided by data on consumer medication information needs.

## Methods

A mixed-method approach was used in this study comprising (1) an assessment of consumer medication information needs, (2) a website content evaluation using the consumer information needs, and (3) user testing of websites (including qualitative interviews).

### Identification of Consumer Medication Information Needs

Consumer medication information needs were determined by examining the most frequently used medications in Australia and the most frequent consumer medication queries made to the healthdirect helpline. The top 5 most commonly used prescription and nonprescription medications in Australia were extracted from the Pharmaceutical Benefits Scheme (PBS) [24] and the National Census of Medicines Use, respectively [25]. The top 5 prescription medications, by defined daily dose/1000 population/day, were atorvastatin, rosuvastatin, perindopril, irbesartan, and candesartan [24]. The top 5 nonprescription medications (excluding paracetamol) used by survey respondents in the past month were fish oil supplements (26.1% of respondents), aspirin (21.5% of respondents), glucosamine (17.5% of respondents), calcium (12.3% of respondents), and cholecalciferol or vitamin D (11.4% of respondents) [25]. Paracetamol (used by 42.9% of respondents in the last month) was excluded from this list as it was the subject of the most frequent consumer medication query made to the healthdirect helpline (see below).

The consumer medication queries made to the healthdirect helpline in November 2014 were extracted and reviewed to determine the medication therapeutic class that was the subject of the call and the query regarding the medication (eg, what to do if a dose was missed). The healthdirect helpline is a free 24-hour health advice telephone line that covers approximately 56% of Australia’s population. The telephone service receives between 60,000 and 70,000 calls per month, with medication queries the most frequent clinical issue discussed [26]. In November 2014, the most frequent medication classes (and specific medication within each class) were analgesics (paracetamol, ibuprofen, and paracetamol and codeine), antibiotics (amoxicillin), antidepressants (sertraline), antihistamines (promethazine), and anticoagulants and antithrombotic agents (warfarin). The most common queries regarding each of these medication classes varied. However, the top 3 queries for all types of medications were (1) how to take a medication (how much to take, what to do if a dose is missed, and what to do in an overdose), (2) medication interactions, and (3) medication side effects.

### Identification of Websites

We aimed to identify websites for the evaluation that consumers would most frequently encounter when using the Google search engine for medication queries. Search terms related to the top medications and queries outlined above were entered into Google. The 4 most frequently generated Australian health websites providing consumer medication advice were selected. Websites specific to a condition, a medication, or a population group were excluded (eg, beyondblue, a website targeting mental health [27]; Royal Children’s Hospital Melbourne targeting pediatric patients [28]). The 4 websites included in our evaluation were Better Health Channel [29], myDr [30], healthdirect [31], and NPS MedicineWise [32].

### Assessment of Website Content

First, each website’s content on the prescription and nonprescription medications identified from the PBS and National Census of Medicines Use was assessed with respect to the extent to which it covered the 3 most frequent medication queries to the healthdirect helpline (ie, how to take a medication, interactions, and side effects). Additionally, whether each website had an information page about the therapeutic classes of these medications was examined. For example, for atorvastatin, websites were evaluated on the extent to which they covered information on how to take the medication (how much to take, what do to if a dose is missed, and what to do in an overdose), interactions, and side effects as well as whether there was a general page on hypolipidemic medications.

Second, each website’s content was assessed with respect to whether it covered the most frequent medication classes that were the subject of calls to the healthdirect helpline and each class’s most frequent queries. Whether each website included information on the most frequent medication within each class was also examined. For example, when providing information on antibiotics, whether the website covered missed doses, interactions, and stopping an antibiotic were assessed, along with whether there was any specific information on amoxicillin. For analgesics and antipyretics, information on paracetamol, ibuprofen, and paracetamol and codeine was examined, as these were by far the most frequent medication types queried and these medicines are available over the counter (nonprescription).

A coding system was applied to indicate the extent to which information on each medication query was available on the websites. Two investigators (MZR and LR) initially tested the coding system to ensure it was suitable and reliable (ie, produced the same code when applied independently by 2 reviewers). Subsequently, a single investigator (LR) coded the queries on all websites. Each query was coded as either covered (C), that is, the information provided on the website was comprehensive enough to fully answer the query; partially covered (PC), that is, there was information related to the query, but it did not answer the query specifically; referred (R), that is, the website referred users to another site that answered the query; and not covered (and not referred; NC). By way of example, for the query “what medications does aspirin interact with?” a website was coded as covering the information if it provided a list of medications with which aspirin interacts but partially covered if it only stated that aspirin interacted with some medications and asked the user to seek advice from a health professional.

Website content assessment was conducted in January 2015.

### User Testing

We developed scenarios for testing based on the medication calls made to the healthdirect helpline outlined above. The most frequently queried medications were combined with the most frequently asked questions to create the scenarios. The scenarios consisted of 8 questions, all of which had answers available on the test websites (Textbox 1). Thus the scenarios sought to test the ease and speed with which users were able to find information that was contained on the websites.

**Scenarios used for website user testing.**

1. Can I take Panadeine Forte (paracetamol 500 mg and codeine phosphate 30 mg per dosing unit) while breastfeeding?

2. It is safe to take my antibiotic (Keflex: cephalexin) with Panadol (paracetamol)?

3. I missed a dose of my antibiotic (Amoxil: amoxicillin), what do I do?

4. What is warfarin (Coumadin) used for?

5. I’m feeling better, can I stop my antibiotic (erythromycin: Eryc)?

6. Is nausea a side effect of my antidepressant (Zoloft: sertraline, a selective serotonin reuptake inhibitor)?

7. Does warfarin interact with Nurofen (ibuprofen, a nonsteroidal anti-inflammatory drug)?

8. Is it safe to take Telfast Decongestant tablets (fexofenadine hydrochloride 60 mg and pseudoephedrine hydrochloride 120 mg per dosing unit) while pregnant?

**Table 1:** Website sequences used for user testing scenarios.

Sequence	Website 1	Website 2	Website 3	Website 4
1	healthdirect	NPS MedicineWise	myDr	Better Health Channel
2	Better Health Channel	healthdirect	NPS MedicineWise	myDr
3	myDr	Better Health Channel	healthdirect	NPS MedicineWise
4	NPS MedicineWise	myDr	Better Health Channel	healthdirect

Consumers who were unfamiliar with the target websites took part in user testing. To recruit participants, posters were displayed at Macquarie University, Sydney campus, Australia. Participants received a complimentary lunch for taking part.

Participants were observed by a single investigator (MTB) while performing the 8 scenario tasks using the websites. To complete each task, the user was required to answer each medication-related query by locating relevant information on a website. Tasks were completed in a fixed order but the order of website use varied between subjects to minimize any learning effects, with each participant randomly allocated to 1 of 4 sequences listed in Table 1. Thus, as there were 8 scenarios, each participant used each website twice to locate a piece of information. User testing was conducted in January and February 2015.

The variables collected by the observer during each scenario were time taken to locate the desired medication information; number of screens required to locate the piece of medication information; number of new searches a user performed (ie, new entries into a search box); the user’s search method (eg, whether he or she used the search box or browsed subheadings); whether the user was successful in completing the task (ie, answered the question correctly); and any obvious negative affect (eg, frustration).

After completion of 4 scenarios (on 2 websites) participants took a short break and were asked to comment on the 2 websites they had just used. They were asked to indicate which website they preferred and why, to describe good and bad features of the websites, and to comment on the layout of information on the screen and on how understandable the website content was. Participants then completed the remaining 4 scenarios and were interviewed about the 2 additional websites. Finally, participants were asked to indicate which of the 4 websites was their preferred website and why and to describe an ideal website for locating medication information.

Nonparametric Friedman tests were used to detect the differences across the websites on time taken to locate information, number of screens required, and number of new searches. A generalized estimating equation approach, with consideration of the correlation of measurements from the same participant, was used to compare the websites on proportion of tasks successfully completed. Results were considered significant when *P* ≤.05.

## Results

### Assessment of Website Content

Table 2 shows website coverage of the most commonly used prescription medications in Australia. Table 3 shows the total percentage of website coverage of the most commonly used prescription medications in Australia. Of the four websites, 3 (NPS MedicineWise, myDr, and Better Health Channel) covered each of the queries related to the medication, and the healthdirect website referred consumers to other sources for the information. All websites had a general information page on hypolipidemic and antihypertensive medications.

Table 4 shows website coverage of the most commonly used nonprescription medications in Australia. Table 5 shows the total percentage of website coverage of information on the most frequently used nonprescription medication in Australia. Compared with prescription medication, information on nonprescription medication was less comprehensive. Whereas all common queries related to these medications were covered by multiple websites for aspirin, calcium, and vitamin D, no website covered all queries on fish oil supplements and glucosamine. Three of the four websites covered general information on both anticoagulants/antithrombotic agents, and complimentary medicines.

**Table 2:** Website coverage of information on the most frequently used prescription medications in Australia.

Prescription medication information		healthdirect	NPS MedicineWise	myDr	Better Health Channel
**Atorvastatin**					
	How to take it^a^	R^b^	C^c^	C	C
	Interactions	R	C	C	C
	Side effects	C^d^	C	C	C
**Rosuvastatin**					
	How to take it^a^	R	C	C	C
	Interactions	R	C	C	C
	Side effects	C^d^	C	C	C
**Perindopril**					
	How to take it^a^	R	C	C	C
	Interactions	R	C	C	C
	Side effects	R	C	C	C
**Irbesartan**					
	How to take it^a^	R	C	C	C
	Interactions	R	C	C	C
	Side effects	R	C	C	C
**Candesartan**					
	How to take it^a^	R	C	C	C
	Interactions	R	C	C	C
	Side effects	R	C	C	C

^a^How to take it includes how much to take, what to do if a dose is missed, and what to do in an overdose. Inclusion of all 3 resulted in a rating of C; if only 1 or 2 items were covered, then a rating of PC (partially covered) was given.

^b^R: referred to an external site.

^c^C: covered.

^d^Covered on a general page about statins, which mentions atorvastatin and rosuvastatin.

**Table 3:** Total percentage of website coverage of information on the most frequently used prescription medications in Australia.

Total coverage (%)		healthdirect	NPS MedicineWise	myDr	Better Health Channel
	Covered	13	100	100	100
	Partially covered	0	0	0	0
	Referred	87	0	0	0
	Not covered	0	0	0	0

**Table 4:** Website coverage of information on most frequently used nonprescription medications in Australia.

Nonprescription medication information		healthdirect	NPS MedicineWise	myDr	Better Health Channel
**Aspirin**					
	How to take it^a^	R^b^	PC^c^	C^d^	C
	Interactions	R	C	C	C
	Side effects	PC	C	C	C
**Fish oil supplements**					
	How to take it^a^	R	PC	NC^e^	PC
	Interactions	R	C	NC	NC
	Side effects	R	C	PC	NC
**Glucosamine**					
	How to take it^a^	R	PC	NC	NC
	Interactions	R	PC	NC	C
	Side effects	R	C	NC	NC
**Calcium**					
	How to take it^a^	R	PC	C	C
	Interactions	R	NC	C	C
	Side effects	R	PC	C	C
**Cholecalciferol (vitamin D)**					
	How to take it^a^	R	PC	PC	C
	Interactions	R	C	NC	C
	Side effects	R	C	C	C

^a^How to take it includes how much to take, what do to if a dose is missed, and what to do in an overdose. Inclusion of all 3 resulted in a rating of C; if only 1 or 2 items were covered then a rating of PC was given.

^b^R: referred to an external site.

^c^PC: partially covered.

^d^C: covered.

^e^NC: not covered (and not referred).

**Table 5:** Total percentage of website coverage of information on the most frequently used nonprescription medications in Australia.

Total coverage (%)		healthdirect	NPS MedicineWise	myDr	Better Health Channel
	Covered	0	47	47	67
	Partially covered	7	47	13	7
	Referred	93	0	0	0
	Not covered	0	6	40	27

Table 6 shows website coverage of the most common queries to the healthdirect helpline related to analgesics and antipyretics. Table 7 shows the total percentage of website coverage of queries to the healthdirect helpline related to simple analgesics and antipyretics. Each query was covered by at least one website for each medication, except for paracetamol and codeine, where no website contained information on whether it is safe to take another dose of paracetamol and codeine after vomiting. No website covered all the queries for paracetamol, and only 1 site (myDr) covered all the queries for ibuprofen.

Table 8 shows the websites’ coverage of the queries that the healthdirect helpline has received related to antibiotics, antidepressants, antihistamines, and anticoagulants and antithrombotic agents. Table 9 shows website coverage of general information queries to the healthdirect helpline related to antibiotics, antidepressants, antihistamines, and anticoagulants and antithrombotic agents. Table 10 shows the total percentage of website coverage of queries to the healthdirect helpline related to antibiotics, antidepressants, antihistamines, and anticoagulants and antithrombotic agents. General information on each medication class was available from at least one website, except for antihistamines, with no website providing general information on this drug class. Information on missed doses of antidepressants was not covered by the websites and that on missed doses of antibiotics was only partially covered by 1 website (NPS MedicineWise). The most common queries related to antibiotics, antidepressants, and antihistamines were not all covered by any one site.

**Table 6:** Website coverage of queries to the healthdirect helpline related to simple analgesics and antipyretics.

Queries for analgesics and antipyretics		healthdirect	NPS MedicineWise	myDr	Better Health Channel
**Paracetamol**					
	How much to take (children)	PC^a^	PC	C^b^	PC
	Interactions	PC	C	C	NC^c^
	Overdose	C	C	NC	C
**Ibuprofen**					
	How much to take (children)	PC	PC	C	C
	Interactions	R^d^	C	C	NC
	Overdose	R	NC	C	C
**Paracetamol and codeine**					
	Interactions	PC	PC	PC	C
	Overdose	PC	C	C	C
	Vomited after taking, is it safe to take another dose	NC	NC	NC	NC

^a^PC: partially covered.

^b^C: covered.

^c^NC: not covered (and not referred).

^d^R: referred to an external site.

**Table 7:** Total percentage of website coverage of queries to the healthdirect helpline related to simple analgesics and antipyretics.

Total coverage (%)		healthdirect	NPS MedicineWise	myDr	Better Health Channel
	Covered	11	44	67	56
	Partially covered	56	33	11	11
	Referred	22	0	0	0
	Not covered	11	22	22	33

**Table 8:** Website coverage of queries to the healthdirect helpline related to antibiotics, antidepressants, antihistamines, and anticoagulants and antithrombotic agents.

Queries	healthdirect	NPS MedicineWise	myDr	Better Health Channel
**Antibiotics**				
Interactions	R^a^	PC^b^	PC	NC^c^
How long to take it for	R	C^d^	C	NC
Missed dose	NC	PC	NC	NC
**Antidepressants**				
Interactions	PC	C	PC	NC
Starting and stopping	PC	C	C	C
Missed dose	NC	NC	NC	NC
**Antihistamines**				
How much to take (children)	R	NC	NC	NC
Interactions	R	NC	NC	NC
Use in breastfeeding	R	C^e^	NC	NC
**Anticoagulants, antithrombotic agents**				
Interactions	R	C	C	NC
Side effects	R	C	C	NC

^a^R: referred to an external site.

^b^PC: partially covered.

^c^NC: not covered (and not referred).

^d^C: covered.

^e^Information on use of antihistamines while breastfeeding was on a page about medication use in breastfeeding.

**Table 9:** Website coverage of general information queries to the healthdirect helpline related to antibiotics, antidepressants, antihistamines, and anticoagulants and antithrombotic agents.

Queries	healthdirect	NPS MedicineWise	myDr	Better Health Channel
**Antibiotics**				
General information?	Yes	Yes	Yes	No
Specific information on amoxicillin?	No	Yes	Yes	Yes
**Antidepressants**				
General information?	Yes	Yes	Yes	Yes
Specific information on sertraline?	No	Yes	Yes	Yes
**Antihistamines**				
General information?	No	No	No	No
Specific information on promethazine?	No	Yes	Yes	Yes
**Anticoagulants, antithrombotic agents**				
General information?	Yes^a^	Yes	Yes	No
Specific information on warfarin?	Yes^a^	Yes	Yes	No

^a^Information provided on a page about stroke treatment.

**Table 10:** Total percentage of website coverage of queries to the healthdirect helpline related to antibiotics, antidepressants, antihistamines, and anticoagulants and antithrombotic agents.

Total coverage (%)		healthdirect	NPS MedicineWise	myDr	Better Health Channel
	Covered	0	55	36	9
	Partially covered	18	18	18	0
	Referred	64	0	0	0
	Not covered	18	27	45	91

### User Testing

A total of 16 consumers were recruited for user testing. The median age was 27 years (range 18-66) and 8 out of 16 participants (50%) were male. When asked how frequently they used the Internet, all participants reported that they used the Internet “multiple times a day.” When asked how often they used the Internet to find medication information, typical responses were “never” (n=7), “rarely” (n=4), and “once per month” (n=2).

Table 11 shows results of user testing. The number of screens viewed while completing scenarios using NPS MedicineWise and myDr were fewer than those viewed when using healthdirect and Better Health Channel (Friedman's χ^2^_3_=9.02, *P*=.03). However, there was no evidence to indicate differences in the time taken to complete scenarios (Friedman's χ^2^_3_=5.47, *P*=.14), the number of new searches performed (Friedman's χ^2^_3_=3.04, *P*=.39), or in the accuracy with which participants completed the scenarios (χ^2^_3_=1.34, *P*=.72).

Of the 16 participants, 11 participants (69%) indicated NPS MedicineWise was their preferred website, 4 participants (25%) said myDr, and 1 participant preferred healthdirect to the other websites.

**Table 11:** Results of scenario-based user testing of websites.

Key results	healthdirect	NPS MedicineWise	myDr	Better Health Channel
Median time taken to complete a scenario (range)^a^	3 min^b^ 37 s^c^ (1 min 13s to 16 min 3s)	2 min 56s (1 min 12s to 8 min 56s)	2 min 41s (49s to 9 min 16s)	4 min 34s (1 min 47s to 12 min 31s)
Median number of screens viewed (range)	10 (3-34)	7 (2-17)	7 (3-15)	10 (5-17)
Median number of new searches (range)^a^	2 (0-7)	0 (0-4)	2 (0-6)	2 (0-9)
Percentage correct^a^	75%	75%	81%	69%

^a^ No evidence to indicate differences between websites.

^b^ min: minute.

^c^ s: second.

Overall, it appeared to be more difficult for participants to locate the appropriate page of information for each scenario than to find the relevant piece of information on a page. In 84 of the 128 (65.6%) scenarios observed, participants began the scenario by entering a keyword or keywords into the home page search box. In 32 of the 128 scenarios (25.0%), participants looked for the website’s medicine page before searching for a particular medication name. When trying to locate a piece of information on a webpage, more than half the participants (n=10) used a keyboard shortcut (ie, control-F) to find a keyword (eg, pregnancy) on the page, whereas the remaining participants scrolled through the information.

Participant interviews provided further information on their perceptions of the usability of websites. All participants reported that information displayed on websites was presented at the right level of difficultly, although some suggested that content was understandable because they were students or researchers:

I think there would be a not insignificant proportion of the population that would struggle with it, because they would baulk at the terminology that’s used.

P11

The inability to search using combinations of search terms (eg, Panadeine Forte AND pregnancy) was identified to be a negative aspect of websites by participants:

Yeah, so if it would work more like Google where I would type in the keywords of what I was looking for then that would be much easier. That was something that I noticed all the websites didn’t do.

P1

Users also reported that being directed to PDF versions of consumer medicines information (CMI) leaflets was problematic:

It’s really wordy and the format of it, because it’s set up, to me, it’s set up like a physical pamphlet, so if I had that in my hands, that’s fine but on the screen, the three column thing with the same format and the font of a physical pamphlet doesn’t work...it makes skimming much harder because, I mean, I could do it but it just took me longer.

P4

A large number of menus and drop-down menus on the home page resulted in participants using the search box rather than browsing the website:

Yeah, so it’s very all over the place really. You really don’t know where to start with that one so you’re almost forced to go to search this side.

P10

The listing of medications only by generic names or brand names, not both, was identified as a barrier to finding relevant information, as users were not necessarily familiar with both terms:

It can be confusing with generic versus brand names, because I know one, when that I was initially looking for Zoloft I looked at that list of anti-depressants and I think they only had the generic names so I didn’t see Zoloft on that list, so I think it’s important to have both written.

P3

Websites presenting medication information in a separate location from health information was perceived as a problem because users generally viewed medication information as a subset of health information, not as a separate category of information:

I think it should be more integrated because I think that that’s logically how people think. They see health as being the generic term and medicine a subset within.

P11

The large number of results being generated from search-box queries was also considered a barrier to locating information:

It’s like when you’re searching, it kind of gives you every possible result rather than the one you probably want, the common one. Well, I don’t know, I mean, it’s hard to get that balance between only throwing up a few common ones, and the person could miss out on what they want to see, or throwing up everything and the person just gets, like, what is all this?

P16

Participants viewed features of websites that broke up large amounts of text (eg, subheadings, highlighting, or hyperlinked subheadings) as helpful:

Subheadings are very good. Especially when you know what you are looking for.

P1

I think that in terms of NPS [MedicineWise] there was more bold so I found it easier to read because then I would just skim and if the bold didn’t apply then I would just ignore the regular font.

P4

Bullet points are good. I mean, you don't want massive slabs of information that you need to search through.

P5

You don’t want to sit there reading through it all. Having those little jumping links is helpful if you are looking for a particular bit of information.

P15

Additionally, auto-completion of search terms in search boxes was reported to be a positive feature of websites:

I like that if you search something there are suggestions for what you are searching.

P6

## Discussion

### Principal Findings

Several limitations were identified in the medication information available on 4 Australian health websites in relation to both content and usability. Although detailed information on specific prescription medication was provided, information on nonprescription medication and medication classes was less comprehensive. Several website features affected how quickly and easily users were able to locate medication information.

Information on the most common prescription medications and most frequent medication queries made to the healthdirect helpline were covered or referred by all 4 websites. The healthdirect website was the only website to refer consumers to other websites. This is because the healthdirect website acts as a portal site that directs consumers to other sources of reliable health information. The majority of the prescription medication information was available through CMI leaflets, either embedded into webpages (NPS MedicineWise, myDr) or as a link to a PDF file (Better Health Channel). CMI content is regulated by the Australian Government and is prepared by and the responsibility of pharmaceutical companies [33]. Although CMI leaflets provide consumers with the full scope of information on a specific medication, the amount of information they present may be overwhelming for consumers [5,33]. Furthermore, CMI leaflets have been criticized for not promoting medication adherence because they include only limited information on the benefits of taking medications [33]. Thus, although it is not feasible for a website to develop its own content on every prescription medication, the inclusion of general pages on medication classes may be an opportunity to provide consumers with more concise information than CMI leaflets, including content on the benefits of taking a medication [5]. In this study, we found the general information pages on 2 prescription medication classes, antibiotics and antidepressants, to be limited in their scope on all websites for answering frequent consumer medication queries.

The way CMI content is displayed on the websites also appeared to be problematic. The inclusion of a link to a PDF version of the “paper” leaflet (as on Better Health Channel), which includes three columns of text, was not viewed favorably by consumers. This was primarily because the layout required users to continuously scroll up and down to read the text. Consumers preferred CMI content to be embedded into the webpage in a single column, as done on NPS MedicineWise and myDr. Interestingly, an assessment of consumer needs in relation to printed CMI leaflets also noted that consumers preferred a single-column layout [33].

Information on nonprescription medication was less comprehensively covered on the websites than prescription medication. The nonprescription medication or medication classes examined in this study included paracetamol, ibuprofen, paracetamol and codeine (formulations with codeine≤12 mg/unit), antihistamines, aspirin, fish oil supplements, glucosamine, calcium, and cholecalciferol. Nonprescription medications do not require a CMI leaflet according to Australian regulations. Instructions for use typically appear in or on the packaging. However, as is evident from the large volume of calls made to the healthdirect helpline about these medications, consumers may not always read, keep, or understand packaging instructions, or all the required information may not be provided on packaging instructions.

Of the nonprescription medicines examined in this study, 5 were complementary medicines. Complementary medicines are a subset of nonprescription medicines that can be defined as herbal, natural, or alternative medicines and include vitamins, minerals, herbs, and nutritional supplements. Australian studies estimate that 50% of complementary medicine users also take conventional medicines [34] and more than half of these consumers do not report complementary medicine use to their doctor [34,35]. Of complementary medicine users, 75% are unaware that the products are not tested for quality and safety by the Australian Therapeutic Goods Administration [34]. Yet, our results showed complementary medicines were the least comprehensively covered by the websites evaluated. There appears to be a significant gap in information available to consumers to make informed decisions about their use of these products. This is particularly salient because the quality of Web-based information on complementary medicines is limited [36].

Although the content of websites is important, it is also crucial for information to be easily located. Usability issues related to both website navigation (ie, locating the correct page) and information display (ie, locating information on a page) were identified in this study. A key navigation issue was that websites did not allow users to search using multiple keywords, as is typically the case in search boxes. This caused users to become extremely frustrated and resulted in delays. Information layout was important for locating content on a page, with participants preferring text to be broken up using subheadings, highlighting, or bullet points. These features are in line with those identified in a previous assessment of consumer needs related to printed CMI leaflets [33]. The NPS MedicineWise website was preferred by the majority of user testing participants. The layout of the NPS MedicineWise website was looked upon favorably by participants and was most likely the reason participants were required to navigate through fewer screens to locate information on this website compared with the other websites.

### Limitations

This study had a number of limitations. The assessment of consumer medication information needs was based on calls to a national health helpline and the most commonly used prescription and nonprescription medications. Consumer queries from other sources, such as health professionals (physicians and pharmacists), were not captured. Additionally, there may be important medication safety issues not recognized by consumers but for which there is limited information, and our methods would not have captured these. The accuracy or the readability level of the medication information on the websites was not evaluated as part of this study. However, a recent study assessing the readability of 251 Australian health webpages found that their readability was above the average Australian levels of reading [8]. Thus, clearly, this is also an important consideration for website design. Lastly, the number of participants used for user testing, although likely large enough to detect most issues [37], limited our ability to detect statistically significant differences between websites for indicators tested. Despite these limitations, the study presents an innovative approach to the evaluation of medication information on websites and identified medication information gaps not previously recognized. Addressing these gaps may improve the safe use of medicines in the community.

### Conclusions

This study applied a unique approach, guided by consumer medication information needs, to assess the content and usability of medication information on 4 Australian websites. Several gaps were identified with respect to website content, and several usability issues were identified with respect to navigation and information presentation. Results showed that the 4 Australian websites tested did not provide consumers with comprehensive medication information on both prescription and nonprescription medications in a user-friendly way. Additional content (eg, on nonprescription medication) and some simple redesign of content (eg, single-column text with bullet points) would improve both the content and usability of widely used Australian websites.

## Abbreviations

CMI: consumer medicines information
PBS: Pharmaceutical Benefits Scheme
